# Evaluation of pH and calcium ion release at the simulated external root resorption cavities of teeth obturated with bioceramic sealer

**DOI:** 10.1002/cre2.573

**Published:** 2022-04-06

**Authors:** Vitchayapun Angkasuvan, Anchana Panichuttra, Mettachit Nawachinda, Chootima Ratisoontorn

**Affiliations:** ^1^ Department of Operative Dentistry, Division of Endodontics, Faculty of Dentistry Chulalongkorn University Bangkok Thailand; ^2^ Department of Restorative Dentistry, Faculty of Dentistry Naresuan University Phitsanulok Thailand

**Keywords:** bioceramic sealer, calcium ion, external root resorption, pH

## Abstract

**Objective:**

The purpose of this study was to evaluate pH and calcium ion release at the outer dentin surface of simulated external root resorption cavities after root canals obturated with bioceramic root canal sealer compared with those medicated with calcium hydroxide.

**Materials and Methods:**

Sixty extracted human single‐rooted teeth were selected and instrumented. External root resorption cavities were prepared at the lingual surface of the root. The teeth were randomly divided into three groups: (1) Bioceramic sealer group, canals were obturated with gutta‐percha and BioRoot sealer; (2) Calcium hydroxide group, canals were medicated with UltraCal XS; (3) Control group, canals were left empty. Thirty specimens were used for evaluation of pH at 7, 14, and 28 days (*n* = 10 per group) and the other 30 specimens were used for evaluation of calcium ion diffusion at 28 days (*n* = 10 per group).

**Results:**

Calcium hydroxide group showed the highest median pH value at all time points (7, 14, and 28 days). Both calcium hydroxide and bioceramic sealer groups showed significantly higher median pH values compared with control (*p* < .001). Comparing within groups, both bioceramic sealer group and calcium hydroxide group showed significantly decreased median pH over time, while the median pH of the control did not show any significant difference among Days 7, 14, and 28. Both calcium hydroxide and bioceramic sealer groups had significantly higher calcium ion release than control. Notably, bioceramic sealer group showed significantly higher calcium ion release than the calcium hydroxide group (*p* < .01).

**Conclusions:**

Root canals obturated with gutta‐percha and bioceramic sealer showed high calcium ion levels at the simulated external root resorption cavities but did not show an extended period of alkaline pH.

## 1 INTRODUCTION

External inflammatory root resorption resulting from traumatic dental injuries is a condition with a potentially rapid onset and is capable of advancing aggressively. Root dentin may be progressive loss over a short period of time if the tooth is left untreated (Soares et al., 2015).

Treatment of external inflammatory root resorption mainly depends on the removal of the infected tissue in the root canal system (Dumsha & Hovland, 1995). This stops the resorption and provides a conducive condition for healing (Cvek, 1992). Effective chemomechanical debridement of the root canal space offers a remarkable success rate of the root canal treatment as well as inhibits and terminates the external root resorption (Dumsha & Hovland, 1995).

Regarding the recommendation for treatment of external inflammatory root resorption following a traumatic dental injury, the canal should be medicated with calcium hydroxide for at least 3 months (Trope, 2002). After an apparent healing of the lesion and a normal periodontal ligament (PDL) was observed, root canal obturation could be performed. This protocol derived from an in vivo study in dogs (Trope et al., 1995), which found that long‐term calcium hydroxide medication showed better cemental repair than short‐term medication due to an alkaline environment. Although relatively insoluble in water, a small portion of calcium hydroxide dissolves and releases calcium and hydroxyl ions. The alkaline environment from hydroxyl ions inhibits osteoclast activity (Bushinsky, 1996; Geng et al., 2009) and activates alkaline phosphatases (Ross et al., 1951), which stops the resorption process and play a significant role in hard tissue formation. While, calcium ions not only activate calcium‐dependent adenosine triphosphate reaction related to hard tissue formation (Estrela et al., 1995) but also take parts in immunologic reactions (Diamantstein & Odenwald, 1974).

Although long‐term calcium hydroxide medication provides efficient management of external inflammatory root resorption (Trope et al., 1995), there are some disadvantages within this treatment regimen. The treatment needs several visits over an extended period of time as well as patient motivation and cooperation. Without patient compliance, the root canal could get infected and cause external root resorption to progress.

In recent years, bioceramic sealers gain popularity because of their physical and biological properties. A report showed that the pH of a bioceramic sealer (BioRoot™ sealer; Septodont, Lancaster, PA, USA), stayed at 12.7 up to 28 days in physiological solution (Khalil et al., 2016).

Studies showed that both hydroxyl ions and calcium ions in calcium hydroxide pastes can spread through the dentinal tubules (Çalt et al., 1999). Accordingly, if hydroxyl ions and calcium ions from BioRoot sealer can disperse from root canal toward the external root surface in an adequate amount over an extended period of time, using long‐term calcium hydroxide medication may be unnecessary. The purpose of this study was to evaluate pH and calcium ion release at the outer dentin surface of simulated external root resorption cavities after root canals obturated with bioceramic root canal sealer compared with those medicated with calcium hydroxide.

## 2 MATERIALS AND METHODS

### 2.1 Sample preparation

The study protocol was approved by the ethics committee of the Faculty of Dentistry, Chulalongkorn University, Bangkok, Thailand (Approval No. 059/2018). A total of 60 extracted human permanent single‐rooted teeth were collected from patients aged 20–34 years old (Carrigan et al., 1984) due to caries, periodontal or orthodontic reasons with informed consent. The teeth were stored in 0.1% thymol solution prior to the experiment. Radiographs were taken to evaluate root dimensions and canal morphology. Teeth that were similar in root shape and root canal size were collected together and equally allocated to each experimental group. Teeth with endodontically treated, resorption, incomplete root formation, or root fracture, were not included in this study.

**Figure 1:**
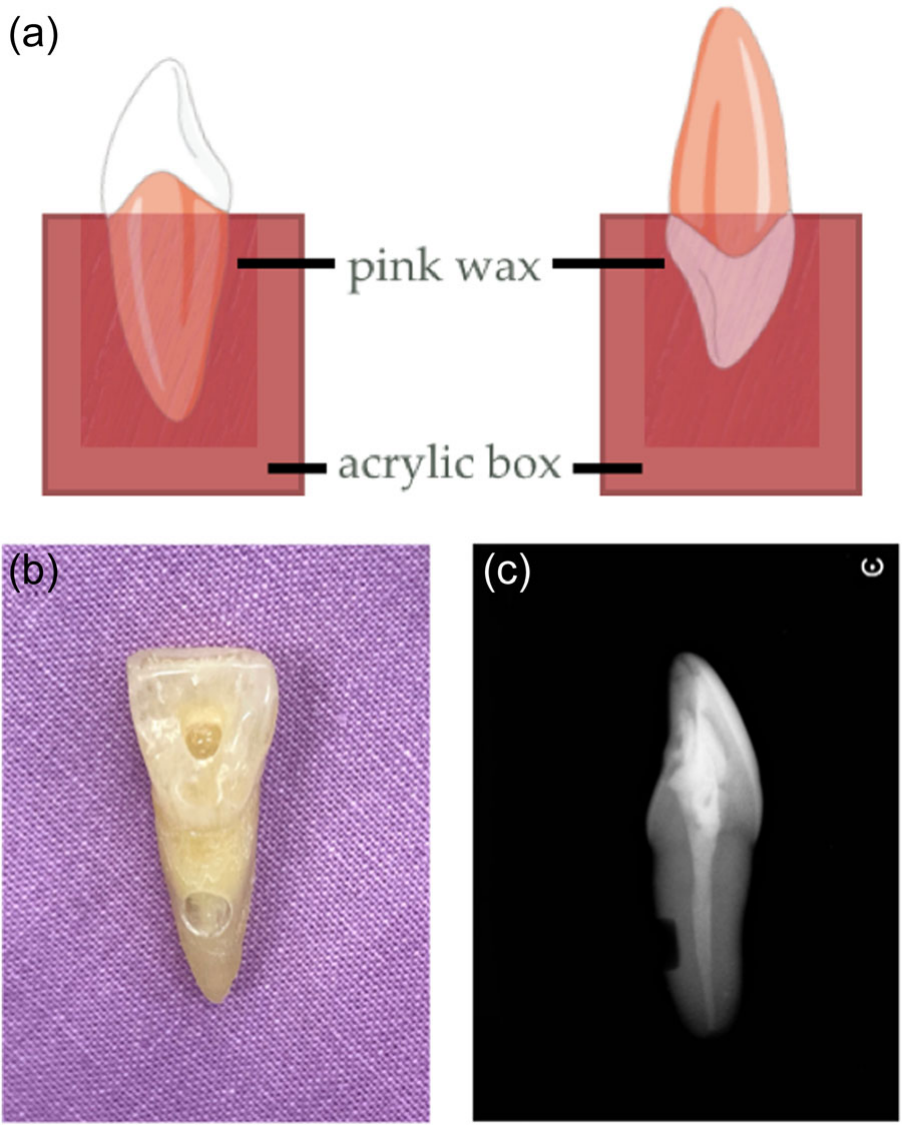
(a) Diagram of acrylic box and pink wax used for holding a tooth during access cavity preparation and root canal preparation (left) and during simulated external root resorption cavity preparation (right); (b) Simulated external root resorption cavity with 3 mm in diameter on the lingual root surface of a prepared sample; (c) Proximal radiograph showed the remaining dentin thickness of 1 mm of the simulated external root resorption cavity

The access cavities were prepared using high‐speed #2 or #4 round burs under cooling water. During access cavity and root canal preparation, the root was fixed in an acrylic box with pink wax as shown in Figure 1a. The total length of the tooth was determined by inserting a size 10 K‐file into the canal until it was visible at the apical foramen. After preparation of the access cavity, a rubber dam was placed to prevent any contamination from sodium hypochlorite to the external root surface. Root canals were instrumented 0.5 mm less than the total length using ProtaperNext (Dentsply Maillefer, Ballaigues, Switzerland) up to three times. Irrigation was performed throughout cleaning and shaping using 5 ml of 2.5% sodium hypochlorite (NaOCl). Lastly, the root canal was flushed with 5 ml of 17% ethylene diamine tetraacetic acid (EDTA) for 1 min to remove the smear layer, followed by 5 ml of 2.5% NaOCl and dried with paper points.

For preparing the external root resorption cavity, the crown was fixed in the acrylic box using pink wax as shown in Figure 1a. Simulated external root resorption cavities sized 3 mm in diameter and remaining dentin thickness of 1 mm were prepared using a high‐speed handpiece with coolant on the lingual surface of the specimen at 5 mm from the apex using cylinder diamond bur (Figure 1b). A custom made putty silicone (Aquasil Soft Putty and Aquasil Ultra LV; Dentsply DeTrey GmbH, Konstanz, Germany) jig for each tooth was used to create a paralleling proximal image for evaluation of the remaining thickness of root canal wall. The remaining dentin thickness of 1 mm was measured from the proximal radiograph using Infinitt program (Infinitt PACS; invented by Infinitt Co., Seoul, Korea) (Figure 1c). The prepared cavity was examined under microscope to make sure that there was no crack in the external root resorption cavity. After preparation, the cavity was rinsed with 1 ml of 17% EDTA for 1 min, followed by 5 ml of distilled water. Teeth were dried and stored at 37°C for 12 h. The apical areas were sealed with sticky wax and varnish coat (Super Top Speed; Revlon, New York, NY, USA) was applied throughout the external root surface excluding the prepared cavities as well as access opening and let to dry for 24 h at room temperature (25°C). At the end of the external root resorption cavity preparation process, we found that only maxillary central incisors, maxillary canines, mandibular canines, and mandibular premolars were used in the experiments. Observingly, maxillary lateral incisors and mandibular central and lateral incisors were not included in this study because the external root resorption cavity cannot be properly prepared to achieve the dimension of the simulated cavity designed for the experiments.

Samples were divided equally into three groups (*n* = 20 each) as follows:

1. UltraCal group: the root canals were filled with UltraCal® XS (Ultradent Products Inc., South Jordan, UT, USA) using lenturo spiral filler size 2 (blue) (Dentsply Maillefer, Tulsa, OK, USA) with slow speed handpiece to the working length;
2. BioRoot group: the root canals were filled with gutta‐percha and BioRoot™ sealer (Septodont, Lancaster, PA, USA) using single cone technic as recommended by the manufacturer; and
3. Control group: the root canals were left empty.

Ultimately, all the samples were sealed coronally with Cavit. Samples of each group were subdivided for pH analysis (*n* = 10) and calcium ion measurement (*n* = 10). Before measurements, a lab assistant randomly numbered 1–30 on the samples pH samples and 1–30 for the calcium ion samples. The operator was blinded to the groups during measurements.

### 2.2 pH measurement

Each sample was immersed only from CEJ to apex in 300 μl of deionized water in a separate vial and stored at 37°C with 100% humidity. pH of the immersion solutions was measured using the pH meter (420A pH meter; Thermo Orion, Inc., Beverly, MA, USA) with microelectrode (InLab® Micro; Mettler Toledo, Greifensee, Switzerland) at 7, 14, and 28 days. After each measurement, the immersion solutions were replaced with fresh 300 μl of deionized water in each vial.

### 2.3 Calcium ion measurement

Each sample was immersed from CEJ to apex in 5 ml of deionized water in a separate vial and stored at 37°C with 100% humidity. After immersion for 28 days, calcium ions were measured by passing immersion media through inductively coupled plasma optical emission spectrometry (ICP‐OES) (ICP‐OES Optima 7300 DV; Perkin Elmer®, Waltham, MA, USA).

### 2.4 Statistical analysis

Data were analyzed using IBM SPSS Statistics for Windows, Version 22.0 (IBM, Armonk, NY, USA). Normally distributed data are presented in mean and standard deviation. Skewed data presented in median and interquartile range. pH values were analyzed using the Kruskal–Wallis test, followed by pairwise comparisons with the Mann–Whitney *U* test. The significance threshold was set to 0.005 (0.05/9), according to Bonferroni's multiple testing correction. Friedman test was used to compare pH within groups at different time points. Calcium ion release data were analyzed by one‐way analysis of variance, followed by Games–Howell post hoc analysis. A *p* < .05 was considered statistically significant.

## 3 RESULTS

### 3.1 pH

The pH values were presented in Table 1. UltraCal group showed the highest median pH values than other groups at 7, 14, and 28 days. Both UltraCal and BioRoot groups showed significantly higher median pH compared to the control group at any point in time. Additionally, UltraCal and BioRoot groups showed no significant different median pH values at 7 days after Bonferroni correction (*p* = .014). While there were significantly different median pH values at 14 and 28 days between UltraCal and BioRoot groups after Bonferroni correction (*p* < .001).

**Table 1:** Median pH (interquartile range) at various time intervals

Groups	7 days	14 days	28 days
UltraCal	11.31 (0.85)^aA^	9.86 (1.28)^aB^	9.31 (1.24)^aC^
BioRoot	10.97 (1.12)^aA^	7.81 (0.63)^bB^	7.21 (0.18)^bC^
Control	6.99 (0.05)^bA^	7.00 (0.06)^cA^	7.01 (0.03)^cA^

*Note*: Same lower case showed no statistically significant difference in the same column. Same upper case showed no statistically significant difference in the same row. Kruskal–Wallis test, followed by pairwise comparisons with Mann–Whitney *U* test, was used to compare pH between different groups at each time points. Significant threshold was set to 0.005 (0.05/9), according to Bonferroni's multiple testing correction. Friedman test, followed by pairwise comparisons with Wilcoxon signed‐rank test, was used to compare pH within groups between different time points.

Comparing within groups, median pH of the control group did not show any significant difference among Days 7, 14, and 28 (*p* > .05). However, in both BioRoot and UltraCal groups, median pH significantly decreased over time (*p* = .05).

### 3.2 Calcium ion release

After immersion for 28 days, both UltraCal and BioRoot groups had significantly higher calcium ion release than the control group. Notably, the BioRoot group showed significantly higher calcium ion release than the UltraCal group as shown in Table 2.

**Table 2:** Mean (standard deviation) of calcium ion release through simulated external root resorption

Calcium ion release	ppm
UltraCal	123.14 (5.59)^a^
BioRoot	248.61 (17.91)^b^
Control	56.03 (7.41)^c^

*Note*: Different superscript letters show statistically significant differences (*p* < .01). Calcium ion release data were analyzed by one‐way analysis of variance, followed by Games–Howell post hoc analysis. A *p* < .05 was considered statistically significant.

## 4 DISCUSSION

Calcium hydroxide paste is frequently used in endodontic treatment to get rid of the infection and cease the resorption process. UltraCal XS is a premixed calcium hydroxide paste, consisting of 35% calcium hydroxide by weight. Diffusion of hydroxyl ions from the root canal through dentinal tubules counterbalances the acidic microenvironment of the resorbed areas attributable to the osteoclast activity (Esberard et al., 1996). In addition, diffusion of calcium ions to the external root surface may be useful for hard tissue formation (Tronstad et al., 1981). Using calcium hydroxide paste as an intracanal medicament significantly increased the pH of dentin (Tronstad et al., 1981), as well as calcium ion level at the external root surface (Foster et al., 1993).

Dentine permeability is directly correlated with the diameter and number of dentinal tubules (Outhwaite et al., 1976). These aspects of the tubules depend not only on their location within the dentin but also on the age of the patient (Carrigan et al., 1984). Accordingly, this study used simulated root resorption cavities at 5 mm from the apices in permanent single‐rooted teeth which were collected from patients 20–34 years of age. In addition, varying dentin thickness (Tsesis et al., 2005) of simulated root resorption cavities could make a difference in pH changes. Therefore, this study prepared samples with the remaining dentin thickness of 1 mm which was confirmed by radiographic examination. This model provided a justified surface area for studying the diffusion of ions through dentinal tubules of the root and reduced differences between samples. Moreover, to ensure that hydroxyl ions and calcium ions diffused to the external root surface through the dentin at the simulated root resorption cavity, the apical areas were sealed with sticky wax and double coats of nail polish were applied on the root thoroughly except the resorption fields (Caicedo et al., 2004; Viapiana et al., 2016). Although it is inconsistent whether removal of smear layer affects hydroxyl ion diffusion through dentinal tubules (Foster et al., 1993; Saif et al., 2008), this study removed the smear layer from both the instrumented intracanal walls and the external root cavities, in order to create the similarities between samples.

After placing calcium hydroxide into a root canal, hydroxyl ions can diffuse into the inner root dentin within hours (Nerwich et al., 1993). However, to get to the outer root dentin, they take 1–7 days and 2–3 weeks to reach the highest level. To attain the effective therapeutic benefit, intracanal dressing with calcium hydroxide for 4 weeks is recommended to arrest external inflammatory root resorption (Nerwich et al., 1993). This study measured pH of immersion solution at 7, 14, and 28 days during the experimental period similar to a previous study (Dudeja et al., 2015). After measuring the pH of the immersion solution at each time point, we replaced the immersion solution to prevent the saturation of the solution. When equilibrium is reached, at which point, no further diffusion takes place. In addition, the roots were immersed in the solution in a closed system. If the seal was broken, air could trap in the solution and carbon dioxide from the air may cause the solution to become more acidic (Byck, 1932).

The diffusion of an alkaline pH from calcium hydroxide intracanal medication to the simulated external root defects was shown in several studies (Esberard et al., 1996; Nerwich et al., 1993; Tronstad et al., 1981; Tsesis et al., 2005). In this study, the UltraCal group showed the highest pH at 7 days followed by 14 and 28 days which is in agreement with previous studies (Dudeja et al., 2015; Siboni et al., 2017). At any immersion time intervals in this study, the UltraCal group also showed higher pH than the BioRoot group. Although one study reported that pH of BioRoot sealer was 12.7 up to 28 days (Khalil et al., 2016), in the other study, pH of the BioRoot sealer was dropped off over time with direct contact measurement (Heward & Sedgley, 2011). Our results showed that the BioRoot group had the highest pH in 7 days, then gradually decreased at 14 and 28 days, respectively, in accordance with the fact that BioRoot sealer completely sets at 5 h (Heward & Sedgley, 2011). After setting, hydroxyl ions are less likely to diffuse through the root dentin.

Tronstad et al. (1981) demonstrated pH range of 7.4–9.6 in the peripheral dentin after calcium hydroxide has been placed in the canals. They also suggested that calcium hydroxide arrested inflammatory root resorption and stimulate healing. The results of this study showed that BioRoot sealer at 7 and 14 days (10.77 + 0.56, 7.76 + 0.53) was within the pH range that could arrest inflammatory root resorption. However, the pH went below the effective range to cease inflammatory root resorption at 28 days (7.26 + 0.20).

Apart from the effect of the high pH of calcium hydroxide, the presence of calcium ions is suggested to be crucial for the activity of the complement system in the immunologic reaction (Morgan & Campbell, 1985). Plenty of calcium ions might support this reaction locally and might also activate a calcium‐dependent ATPase, which has been associated with hard tissue formation (Magnusson & Linde, 1974). Nonetheless, the manufacturer did not declare the calcium content of BioRoot sealer. High calcium ion detected from direct contact measurement of BioRoot sealer was reported in previous studies (Khalil et al., 2016, Xuereb et al., 2015). Calcium level of the soaking solution exposed to BioRoot sealer for 14 days was 29,712 mg/g (Xuereb et al., 2015) and for 28 days was 28,682 mg/g (Khalil et al., 2016). In this study, we evaluated calcium ion release at the outer dentin surface of the simulated external root resorption cavity after obturation with BioRoot sealer. Therefore, we found a lower amount of calcium ion release (248.61 + 17.91 ppm) than previous studies (Khalil et al., 2016; Xuereb et al., 2015).

A previous report showed that UltraCal XS had low calcium ion release (1.2206 ppm) at the outer dentin surface of simulated external root resorption cavity after medication for 28 days (Dudeja et al., 2015). Nonetheless, our study showed that calcium ion release in the UltraCal group (123.14 + 5.59 ppm) was higher than in the previous study (Dudeja et al., 2015). This can be explained by the larger size of the simulated root cavities in this study. Although there is no study that directly compared calcium ion release from BioRoot sealer with UltraCal XS, Xuereb et al. (2015) found that calcium ion release from BioRoot sealer was extremely higher than MTA sealer and Dudeja et al., (2015) found that calcium ion release from MTA sealer was higher than UltraCal XS. We may infer from this evidence that BioRoot sealer has higher calcium ion release when compare with UltraCal XS, which is in agreement with the results of this study.

The solubility measurement showed that BioRoot sealer had less than 3% weight loss which meets the ISO standard (ISO 6876) (Prüllage et al., 2016), but it nonetheless had high solubility before setting and the solubility was significantly lower after setting (Heward & Sedgley, 2011). It is possible that the presetting sealer released a high amount of hydroxyl ions. After the sealer was set, hydroxyl ions were less available. Likewise, our study showed high alkaline pH at the first measurement (7 days), which included the presetting period, then the pH declined after the material was set.

High calcium ions released from the BioRoot sealer might be useful to inhibit root resorption and promote PDL healing. The number of calcium ions affects osteoclast activity (Zaidi et al., 1999). Free cytosolic calcium was rapidly increased after osteoclasts were exposed to unusually high calcium ions concentrations (5–20 mM) which leads to a reduction of bone resorption activity and enzyme release (Zaidi et al., 1999). Furthermore, the combination of 9.0 mM calcium ions and 4.5 mM phosphate ions could significantly influence the proliferation, differentiation, and mineralization of human periodontal ligament cells (An et al., 2012). The equivalent concentration range of calcium ions also promoted cell proliferation of human adipose‐derived stem cells and mouse osteoblasts (Maeno et al., 2005; McCullen et al., 2010; Nakamura et al., 2010).

Even though the results of this study indicate that when BioRoot sealers are used during obturation, the pH at the surface of the root is not as high as those of root medicated with calcium hydroxide (Tronstad et al., 1981) over an extended period of time, an abundance of calcium ion release at the root surface might promote the arresting and healing on inflammatory root resorption (An et al., 2012; McCullen et al., 2010; Nakamura et al., 2010; Tronstad et al., 1981; Zaidi et al., 1999). Thus, further research should be performed in vivo to evaluate whether inflammatory root resorption could be controlled by root canal obturation with gutta‐percha and BioRoot sealer.

## 5 CONCLUSION

Although root canal obturated with gutta‐percha and BioRoot sealer showed high calcium ion release, the pH at the external root resorption cavity was lower than that medicated with calcium hydroxide after 7 days. If an extended period of alkaline pH is required for treatment of external inflammatory root resorption, BioRoot sealer could not replace long‐term medication with calcium hydroxide paste for treatment of root resorption.

## AUTHOR CONTRIBUTIONS

Vitchayapun Angkasuvan contributed to study design, specimen preparation, data acquisition, data analysis and interpretation, and draft manuscript. Anchana Panichuttra contributed to critical feedback, discussed the results, and commented on the manuscript. Mettachit Nawachinda contributed to critical feedback, discussed the results, and commented on the manuscript. Chootima Ratisoontorn contributed to project conception, study design, project supervision, and preparation of the manuscript.

## CONFLICTS OF INTEREST

The authors declare no conflicts of interest.

## Data Availability

The data that support the findings of this study are available from the corresponding author upon reasonable request.
